# MiR-365-3p inhibits lung cancer proliferation and migration via CPT1A-mediated fatty acid oxidation

**DOI:** 10.1038/s41598-025-91665-x

**Published:** 2025-02-27

**Authors:** Dan Xu, Bohong Liu, Lingling Wang

**Affiliations:** 1 Beihua University Affiliated Hospital, Jilin, 132013 China; 2 Department of Chest Medicine, Changchun Tumor Hospital, Changchun, 130012 China; 3 Department of Neurology, Beihua University Affiliated Hospital, No. 12 Jiefang Middle Road, Jilin city, 132013 Jilin Province China

**Keywords:** Lung cancer, miR-365-3p, CPT1A, Fatty acid oxidation, Proliferation, Migration, Lung cancer, Cancer metabolism

## Abstract

The relationship between abnormal lipid acid metabolism and the progression of lung cancer is increasingly evident. Carnitine palmitoyltransferase 1A (CPT1A), a rate-limiting enzyme in fatty acid oxidation, has been implicated in the advancement of various cancers. However, the role of CPT1A in lung cancer and the regulatory mechanisms of microRNAs on CPT1A-mediated fatty acid oxidation remain largely unknown. In our study, we demonstrate that miR-365-3p inhibits CPT1A expression by targeting its 3’-untranslated region in lung cancer cells. The inhibition of CPT1A by miR-365-3p leads to increased lipid droplet accumulation, diminished ATP production, and a decrease in fatty acid oxidation levels. Furthermore, the disruption of fatty acid oxidation attenuates the ability of the miR-365-3p/CPT1A axis to modulate lung cancer cell proliferation and migration both in vitro and in vivo. Clinical data reveal that CPT1A expression is significantly upregulated while miR-365-3p is markedly downregulated. Additionally, there exists a negative correlation between miR-365-3p and CPT1A expression, and both are predictive of clinical outcome in lung cancer patients. Collectively, our findings shed light on the function and mechanistic pathway of the miR-365-3p/CPT1A axis in lung cancer, which might provide a potential therapeutic target for lung cancer.

**Supplementary Information:**

The online version contains supplementary material available at 10.1038/s41598-025-91665-x.

## Introduction

Lung cancer is one of the most prevalent cancers worldwide and remains the primary cause of cancer-related mortality^1,2^. Numerous risk factors contribute to lung cancer, including lifestyle choices, environmental exposure, and genetic susceptibilities, in addition to their interactive effects, which are instrumental in tumorigenesis^3,4^. Despite significant improvements in treatment modalities for lung cancer over the past decade, the underlying mechanisms driving the growth and progression of lung cancer remain largely unknown. Consequently, there is an urgent need to investigate the pathogenesis and evolution of lung cancer to pave the way for more effective interventions.

Unrestrained cell proliferation is a hallmark of tumor cells^5^. Compared with normal cells, tumor cells exhibit markedly altered metabolic processes optimizing their energy acquisition to support rapid cell growth^6,7^. Therefore, malignant tumors are considered to be closely associated with energy metabolism. In addition to the well-documented aerobic glycolysis, known as the Warburg effect, dysregulation of fatty acid metabolism is a crucial aspect of tumor metabolic reprogramming^8–10^. Fatty acid oxidation (FAO) plays a significant role in fatty acid metabolism and is frequently activated in various cancers to produce additional energy sources^11^. Carnitine palmitoyltransferase 1 (CPT1) acts as a rate-limiting enzyme in this process, facilitating the transfer of fatty acids into mitochondria for β-oxidation. Among CPT1 isozymes, CPT1A has been implicated in the progression of several cancer types, including colorectal, breast, gastric, and prostate cancers^12–16^. However, the role of CPT1A in lung cancer remains unclear.

Mounting evidence indicates that microRNAs (miRNAs) are pivotal modulators of fatty acid metabolism and contribute to tumor progression^17–19^. These small non-coding RNAs can affect metabolic processes by binding to the 3’-untranslated regions (UTRs) of genes encoding enzymes involved in fatty acid metabolism, thereby modulating their expression. Notably, miR-328-3p has been identified as a regulator of CPT1A, inhibiting FAO and reducing the invasion and metastasis of breast cancer cells^13^. Similarly, miR-377-3p has been shown to curtail the growth and metastasis of hepatocellular carcinoma through CPT1C, subsequently affecting FAO^20^. MiR-107 regulates the expression of CPT1A by targeting, thereby modulating breast cancer growth and metastasis^14^. MiR-132-3p promotes hepatocellular carcinoma growth and metastasis through suppression of fatty-acid oxidation^21^. However, the miRNAs that target CPT1A to regulate CPT1A-mediated FAO in lung cancer remains unknown.

In our study, we found that miR-365-3p targeted CPT1A and effectively reduced its expression. By modulating fatty acid metabolism in lung cancer via the CPT1A-mediated FAO pathway, miR-365-3p plays a critical role in inhibiting the proliferation and migration of cancer cells, both in vivo and in vitro. Thus, the miR-365-3p/CPT1A regulatory axis may be a potential therapeutic target for lung cancer.

## Materials and methods

### Tissue specimens and cell culture

Eighty-three paired lung cancer tissues and adjacent normal tissues were collected from Changchun Tumor Hospital. All pathological specimens were confirmed by experienced pathologists. Tissues were collected during surgical operations and promptly stored in liquid nitrogen. The clinical characteristics of the patients are presented in Table S1. The study was approved by the Institutional Review Committee of Changchun Tumor Hospital, and conducted in accordance with the principles of Declaration of Helsinki. The informed consents were obtained from all participants. Human lung cancer cell lines (A549 and H1299) and the human embryonic kidney cell line HEK293T were purchased from the American Type Culture Collection (Manassas, USA). Cells were cultured in DMEM medium (Gibco, USA) containing 10% FBS at 37℃ with 5% CO_2_.

### Plasmids, transfection, and reagents

MiR-365-3p and other miRNA mimics were synthesized by GenePharma (Shanghai, China). The lentiviral plasmid that expresses CPT1A short hairpin RNA (shRNA) was constructed by cloning CPT1A shRNA fragment into pSIH-H1-Puro (System Biosciences). The sequences are listed in Table S2. Lipofectamine RNAiMAX was used to transfect miR-365-3p mimics according to the manufacturer’s instructions (Invitrogen). Lentiviruses were produced by cotransfection of HEK293T cells with recombinant lentivirus vector and pPACK Packaging Plasmid Mix according to the manufacturer’s instructions (System Biosciences). A549 and H1299 cells were infected with the lentiviruses and selected with 1 µg/ml puromycin to generate stably cell lines.

### Western blot analysis

The cells were harvested, washed with PBS and lysed in RIPA buffer for 30 min on ice. The lysates were separated using sodium dodecyl sulfate-polyacrylamide gel electrophoresis, and transferred to nitrocellulose membranes. Subsequently, the membrane was blocked with 5% skim milk for 1 h, incubated with the specific primary antibody overnight at 4℃, and then followed by secondary antibody incubation for 1 h at room temperature. Finally, the bands were detected using chemiluminescence. Anti-CPT1A (ab220789) was obtained from Abcam, anti-β-actin (sc-47778) was purchased from Santa Cruz, and anti-rabbit horseradish peroxidase-conjugated IgG (A0208) was acquired from Beyotime (Shanghai, China).

### Quantitative real-time PCR (qRT-PCR)

The cells were harvested, washed with PBS, and lysed in TRIzol reagent following the manufacturer’s instructions (Invitrogen). Total RNA was reverse-transcribed into cDNA using the HiScript 1st Strand cDNA Synthesis Kit (Vazyme, China). For miR-365-3p, reverse transcription was performed using an miRNA 1st Strand cDNA Synthesis Kit (Vazyme). qRT-PCR was conducted using qPCR SYBR Green Master Mix according to the manufacturer’s instructions (Vazyme). The relative expression was calculated by the 2^−△△Ct^ method. The relevant primer sequences are listed in Table S2.

### Luciferase activity assay

The cells were seeded in 24-well plates and incubated overnight. Subsequently, the cells were co-transfected with a luciferase reporter vector containing wild-type or mutant CPT1A 3’-UTR, and miR-365-3p mimics or a negative control. After transfection for 48 h, the cells were harvested and analyzed using the Dual Luciferase Reporter Assay Kit, following the manufacturer’s instructions (Vazyme).

### Oil red O staining assay

Oil Red O staining was performed according to the manufacturer’s instructions^22^. In brief, cells were seeded into 24-well plates pre-placed with coverslips. The cells were washed with PBS and fixed with 4% paraformaldehyde for 15 min. Subsequently, the cells were stained with Oil Red O working solution for 15 min, washed with washing solution for 30 s, and counter-stained with hematoxylin for 20 s. Finally, the cells were covered with PBS and photographed under a light microscope.

### Immunofluorescence staining and immunohistochemistry assays

For immunofluorescence staining, cells were seeded in 24-well plates pre-coated with coverslips as described before^23^. Briefly, after washing with PBS, the cells were fixed with 4% paraformaldehyde for 15 min, washed twice with PBS, and stained with BODIPY 493/503 for 15 min at 37℃. Nuclei were stained with DAPI for 5 min at room temperature. Images were captured using a fluorescence microscope.

For immunohistochemistry, miR-365-3p expression was detected using miRNA in situ hybridization in paraffin-embedded tissues. Specific probes labeled with digoxin were applied according to the manufacturer’s instructions (Exonbio, China), and the signal was amplified by TSA Plus Cyanine 5 system (PerkinElmer).

### Fatty acid oxidation (FAO) analysis

The FAO assay was performed according to the manufacturer’s instructions (Abcam, Cambridge, UK). Cells were seeded in 96-well plates and incubated overnight. As a positive control, cells were treated with 2.5 µM FCCP. As a negative control, cells were treated with 40 µM Etomoxir. The slope (m) of the linear portion of each profile was regarded as the FAO rate, which indicates the FAO level. The calculation formula was as follows: FAO = m_untreated_ - m_Etomoxir_.

### ATP production assay

The ATP production assay was conducted according to the manufacturer’s instructions (Beyotime). In brief, the cells or tissues were washed with PBS and lysed in lysis buffer. After centrifugation, the supernatant was added to an ATP detection working solution. Luminescence was measured using a luminometer.

### Cell proliferation assay

The cells were seeded in 96-well plates at 2 × 10^3^ cells/well. A CCK-8 kit was used to detect cell proliferation at the indicated times, according to the manufacturer’s instructions (Dojindo, Japan).

### Wound healing assay

The cells were seeded in six-well plates at a density of 90%. Cells were scratched using a 200-µL pipette tip, washed twice with PBS, and incubated in DMEM medium containing 0.5% FBS. After incubation for 24 h, wound healing distance was measured and analyzed to detect cell migration.

### In vivo nude mice assay

Animal studies were performed in accordance with regulations and guidelines for the care and use of laboratory animals and approved by the Institutional Animal Care and Use Committee at Changchun Tumor Hospital. Male BALB/c mice (6-week-old) were purchased from Vital River Laboratory Animal Technology (Beijing, China). Mice were divided into four groups after one week of acclimatization. Approximately 1 × 10^7^ A549 cells stably expressing CPT1A shRNA or control shRNA in 100 µL PBS were injected subcutaneously into the right flanks of mice. Seven days after injection, 15 µg miR-365-3p or negative control in 50 µL PBS was injected into the tumors at multiple points, twice a week for 4 weeks. The tumor size was measured at the indicated time points. It was critical to ensure that the tumors in the mice did not grow too large, specifically, the diameter of any dimension could not exceed 2 cm. After the experiment, the mice were euthanized using 50% volume displacement per minute of 100% carbon dioxide, in accordance with ARRIVE guidelines to ensure humane and ethical treatment. The excised tumors were frozen in liquid nitrogen for further analysis.

### Statistical analysis

All data were independently repeated three times and presented as the mean ± SD. Data analysis was performed using SPSS 20.0 and GraphPad Prism 7.0. Student’s t-test was used to compare two groups, and one-way analysis of variance (ANOVA) was used to analyze multiple groups. Pearson’s correlation analysis was used to analyze the relationship between CPT1A and miR-365-3p expression. A p-value of *P* < 0.05 was considered statistically significant.

## Results

### miR-365-3p inhibits CPT1A expression by targeting the 3’-UTR of CPT1A mRNA

**Fig. 1:**
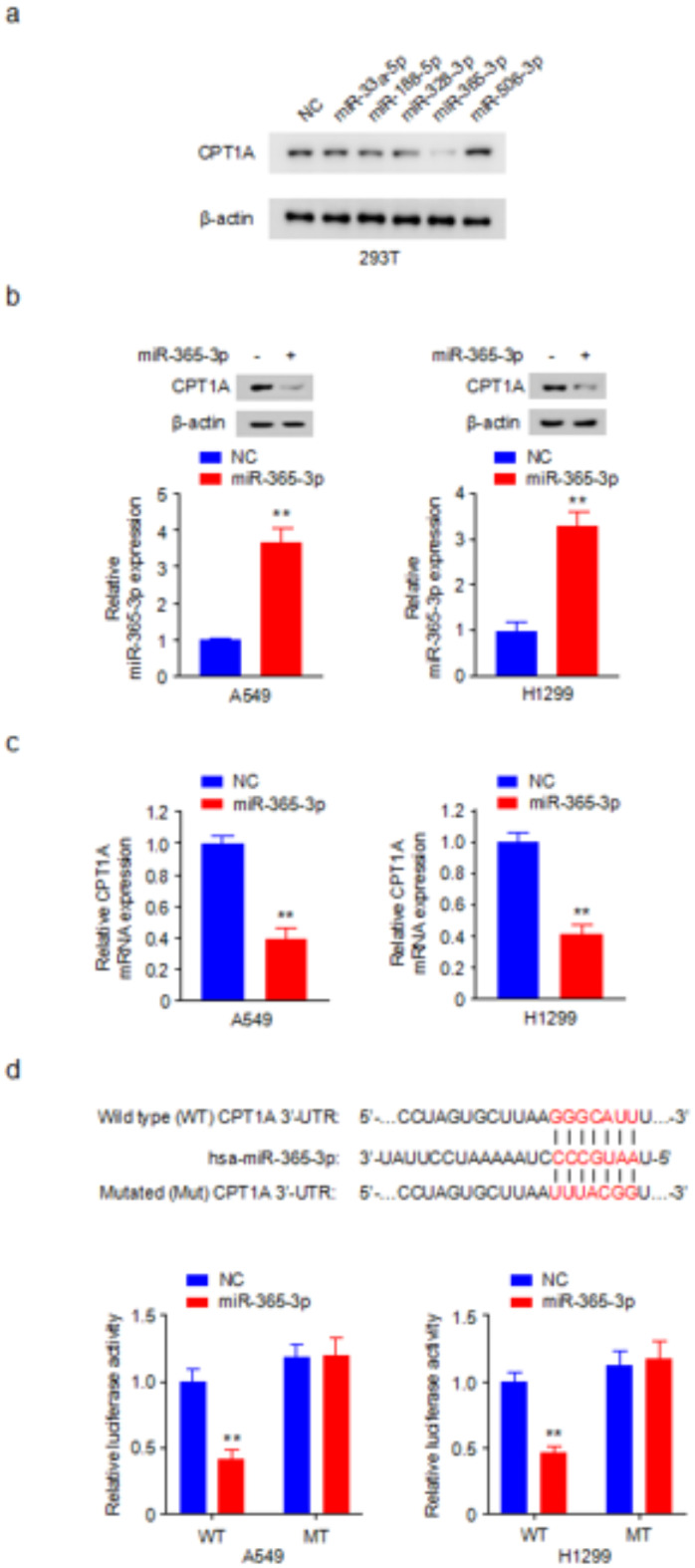
miR-365-3p inhibits CPT1A expression by targeting the 3’-UTR of CPT1A mRNA. (**a**)Western blot analysis of CPT1A protein expression in 293T cells transfected with negative control (NC) or mimics of candidate miRNAs as indicated. β-actin was used as loading control.(**b**)Western blot analysis of CPT1A protein expression in A549 and H1299 cells transfected with NC or miR-365-3p mimics. β-actin was used as loading control. Histograms show relative miR-365-3p expression. (**c**)qRT-PCR detection of CPT1A mRNA expression in A549 and H1299 cells transfected with NC or miR-365-3p mimics. (**d**) Luciferase activity assay was performed in cells co-transfected with CPT1A wild-type 3’-UTR (WT) or mutant 3’-UTR (MT) and NC or miR-365-3p mimics in A549 and H1299 cells. *, *P* < 0.05; **, *P* < 0.01.

Utilizing the predictive power of computational tools, such as TargetScan and miRWalk 2.0, we successfully identified five potential miRNAs as likely candidates for targeting CPT1A: miR-33a-5p, miR-188-5p, miR-328-3p, miR-365-3p, and miR-506-3p. Western blot analysis revealed that miR-365-3p significantly decreased CPT1A expression levels, compared with those of the negative control (NC). By contrast, miR-506-3p slightly promoted CPT1A expression, possibly through an unknown protein. The other candidate miRNAs did not elicit a significant alteration in CPT1A expression levels (Fig. 1a). This finding indicated the potential capability of miR-365-3p to modulate CPT1A expression, thereby miR-365-3p was selected for the further detailed investigation. Consistent with our preliminary observations in 293T cells, the miR-365-3p mimics inhibited CPT1A expression in A549 and H1299 lung cancer cells (Fig. 1b). Moreover, qRT-PCR assay confirmed this effect at the mRNA level, revealing a significant reduction in CPT1A mRNA expression upon transfection with miR-365-3p mimics. Our findings underscored the profound regulatory effect of miR-365-3p on CPT1A at the transcriptional level (Fig. 1c). To explore whether CPT1A was a direct target gene of miR-365-3p, both A549 and H1299 cells were transfected with luciferase reporters linked to either the wild-type or mutated 3’-untranslated region (3’-UTR) of CPT1A, and these cells were also transfected with miR-365-3p mimics or a negative control (NC). Luciferase activity assay results illustrated that miR-365-3p mimics suppressed luciferase activity associated with the wild type 3’-UTR of CPT1A, but did not affect the mutated 3’-UTR luciferase activity (Fig. 1d). Thus, miR-365-3p directly targeted the 3’-UTR region of CPT1A, leading to a decrease in its expression. These experiments conclusively demonstrated that CPT1A is directly targeted by miR-365-3p.

### miR-365-3p suppresses FAO through CPT1A in lung cancer cells

**Fig. 2:**
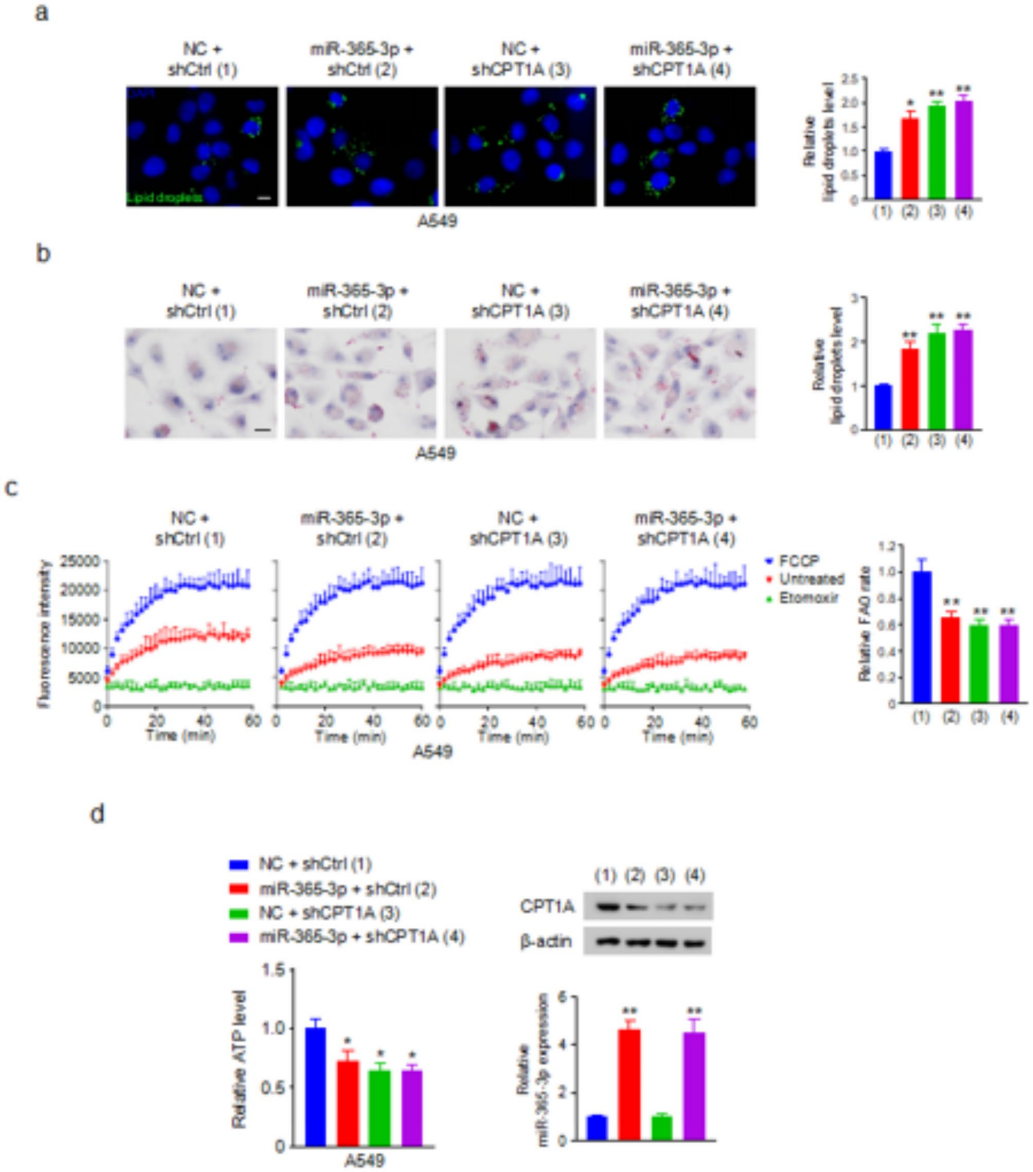
miR-365-3p suppresses FAO through CPT1A in lung cancer cells.(**a**) Immunofluorescence staining to detect the level of lipid droplets (green) in A549 cells stably infected with lentivirus carrying CPT1A shRNA (shCPT1A) or control shRNA (shCtrl) and transfected with miR-365-3p mimics or NC. Scale bar, 10 μm. Histograms show relative lipid droplets level.(**b**) Oil Red O staining assay of A549 cells transfected as in (**a**). Scale bar, 10 μm. Histograms show relative lipid droplets level. (**c**) FAO assay of A549 cells transfected as in (**a**). Cells treated with FCCP or Etomoxir were used as positive and negative controls, respectively. Histograms show relative FAO rate. (**d**) ATP production assay of A549 cells transfected as in (**a**). The representative Western blot shows the expression of CPT1A. qRT-PCR analysis indicates the expression of miR-365-3p. *, *P* < 0.05; **, *P* < 0.01.

As CPT1A plays a critical role in FAO, we explored the hypothesis that miR-365-3p influences FAO in lung cancer cells by modulating CPT1A activity. To track changes in intracellular lipid metabolism, BODIPY 493/503 staining was used to detect lipid droplets within the cells. Immunofluorescence staining revealed that both miR-365-3p mimics and CPT1A knockdown led to a noticeable increase in the quantity of the lipid droplets present in lung cancer cells. Importantly, when CPT1A was knocked down in cells that had already been transfected with miR-365-3p, the ability of miR-365-3p to regulate lipid droplet content was negated (Fig. 2a and Fig. S1a). To confirm these findings, we performed Oil Red O staining assays. Consistent with the BODIPY staining results, Oil Red O staining corroborated the increase in lipid droplets associated with miR-365-3p mimicry and CPT1A knockdown, and this effect was reversed when CPT1A was knocked down in miR-365-3p-transfected cells (Fig. 2b and Fig. S1b). Moreover, FAO analysis demonstrated that the miR-365-3p mimics or CPT1A knockdown reduced the rate of FAO, and knockdown of CPT1A in miR-365-3p-transfected cells abrogated the function of miR-365-3p in A549 and H1299 cells (Fig. 2c and Fig. S1c). In addition to investigating lipid accumulation, this study extended to the evaluation of ATP production. ATP production assays indicated that the presence of miR-365-3p mimics or CPT1A knockdown led to a noticeable decrease in ATP production within lung cancer cells, reinforcing the notion that miR-365-3p regulation of CPT1A has a profound effect on the metabolic processes within lung cancer cells (Fig. 2d and Fig. S1d). Our findings support the hypothesis that miR-365-3p exerts its effects on lung cancer cell metabolism primarily through the repression of CPT1A expression, leading to diminished FAO and consequent alterations in energy production.

### The miR-365-3p/CPT1A axis regulates lung cancer cell proliferation and migration in vivo and in vitro

**Fig. 3:**
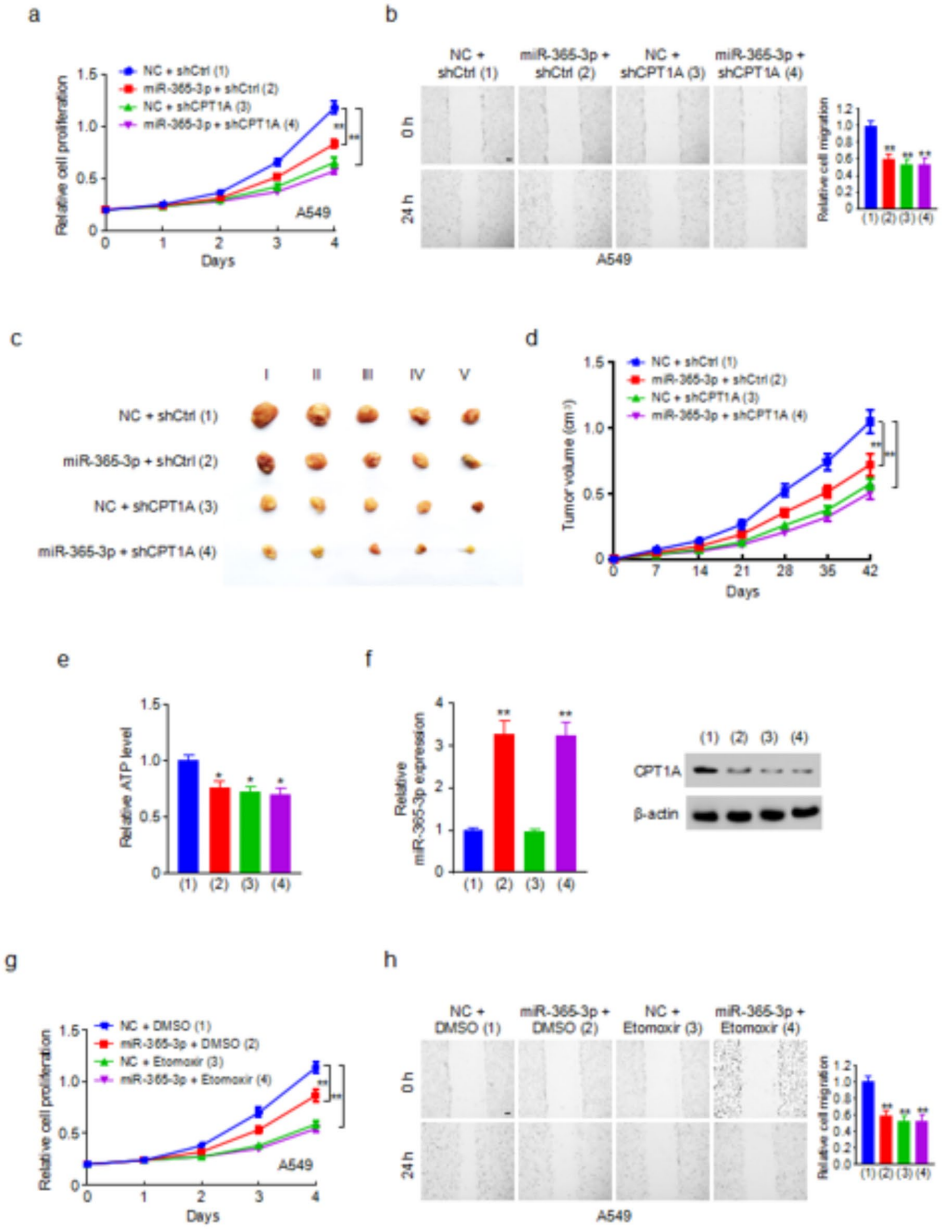
The miR-365-3p/CPT1A axis regulates lung cancer cell proliferation and migration in vivo and in vitro. (**a**) Cell proliferation detection of A549 cells stably infected with lentivirus carrying CPT1A shRNA (shCPT1A) or control shRNA (shCtrl) and transfected with miR-365-3p mimics or NC.(**b**) Wound healing assay of A549 cells transfected as in (**a**). Scale bar, 100 μm. Histograms show relative cell migration.(**c**) A549 cells stably infected with lentivirus carrying shCPT1A or shCtrl were injected into nude mice, and tumors were treated with miR-365-3p or NC as indicated. After 42 days, mice were euthanized to harvest tumors. (**d**)Tumors were measured at the indicated times, and the growth curve was plotted (*n* = 5). (**e**) ATP production of representative tumor tissue (mouse No. III). (**f**) Western blot of representative tumor tissue (mouse No. III) shows the expression of CPT1A. qRT-PCR analysis indicates the expression of miR-365-3p. (**g**) Cell proliferation detection of A549 cells transfected with miR-365-3p mimics or NC and treated with DMSO or Etomoxir (5 µM) as indicated.(**h**)Wound healing assay of A549 cells transfected as in (**g**). Scale bar, 100 μm. Histograms show relative cell migration. *, *P* < 0.05; **, *P* < 0.01.

Given that CPT1A involvement in tumor progression has been established, our study aimed to explore the function of the miR-365-3p/CPT1A axis in influencing lung cancer cell behavior, with a particular focus on cell proliferation and migration capabilities. Cell proliferation assays revealed that both miR-365-3p mimics and CPT1A knockdown significantly reduced the proliferation rates of A549 and H1299 lung cancer cells. Notably, CPT1A knockdown in miR-365-3p-transfected cells almost attenuated the ability of miR-365-3p to modulate cell proliferation (Fig. 3a and Fig. S2a). Additionally, wound healing assays were performed to assess the effect of miR-365-3p and CPT1A manipulation on cell migration. As expected, the wound healing assay indicated that both the miR-365-3p mimics and CPT1A knockdown effectively decreased the migration of A549 and H1299 cells, with minimal effect of the miR-365-3p mimics on cell migration in CPT1A knockdown cells (Fig. 3b and Fig. S2b). Collectively, these findings underscore the critical role of the miR-365-3p/CPT1A axis in modulating lung cancer cell proliferation and migration.

To investigate the role of the miR-365-3p/CPT1A axis in vivo, a subcutaneous injection model was used in male nude mice. A549 cells treated with either a negative control (NC) or miR-365-3p mimics and control shRNA or shRNA targeting CPT1A were injected into the right flank of the mice. Consistent with the results in vitro, the overexpression of miR-365-3p or knockdown of CPT1A effectively suppressed tumor growth (Fig. 3c, d). Importantly, CPT1A knockdown in cells transfected with miR-365-3p almost abolished the ability of miR-365-3p to inhibit cancer xenograft growth (Fig. 3c, d). Further supporting our observations, immunofluorescence staining, ATP production, and western blot assays conducted on the extracted tumor masses revealed that miR-365-3p significantly suppressed FAO by regulating CPT1A (Fig. 3e, f). These findings collectively demonstrate the mechanism by which miR-365-3p suppresses tumor growth, mainly through the inhibition of CPT1A.

Subsequently, we aimed to determine whether the miR-365-3p/CPT1A axis in cell proliferation and migration was mediated through the modulation of FAO. The cell proliferation assay showed that the miR-365-3p-induced reduction in cell proliferation was abolished by treatment with Etomoxir, an inhibitor of CPT1 (Fig. 3g and Fig. S2c). Consistent with these findings, the wound healing assay showed similar trends, the migration inhibition observed with miR-365-3p expression was countered by the application of Etomoxir, highlighting the reliance of this effect on FAO inhibition (Fig. 3h and Fig. S2d). These results underscore the critical role of the miR-365-3p/CPT1A axis in cell proliferation and migration via FAO pathway.

### miR-365-3p is negatively correlated with CPT1A expression and predicts clinical outcome in patients with lung cancer

**Fig. 4:**
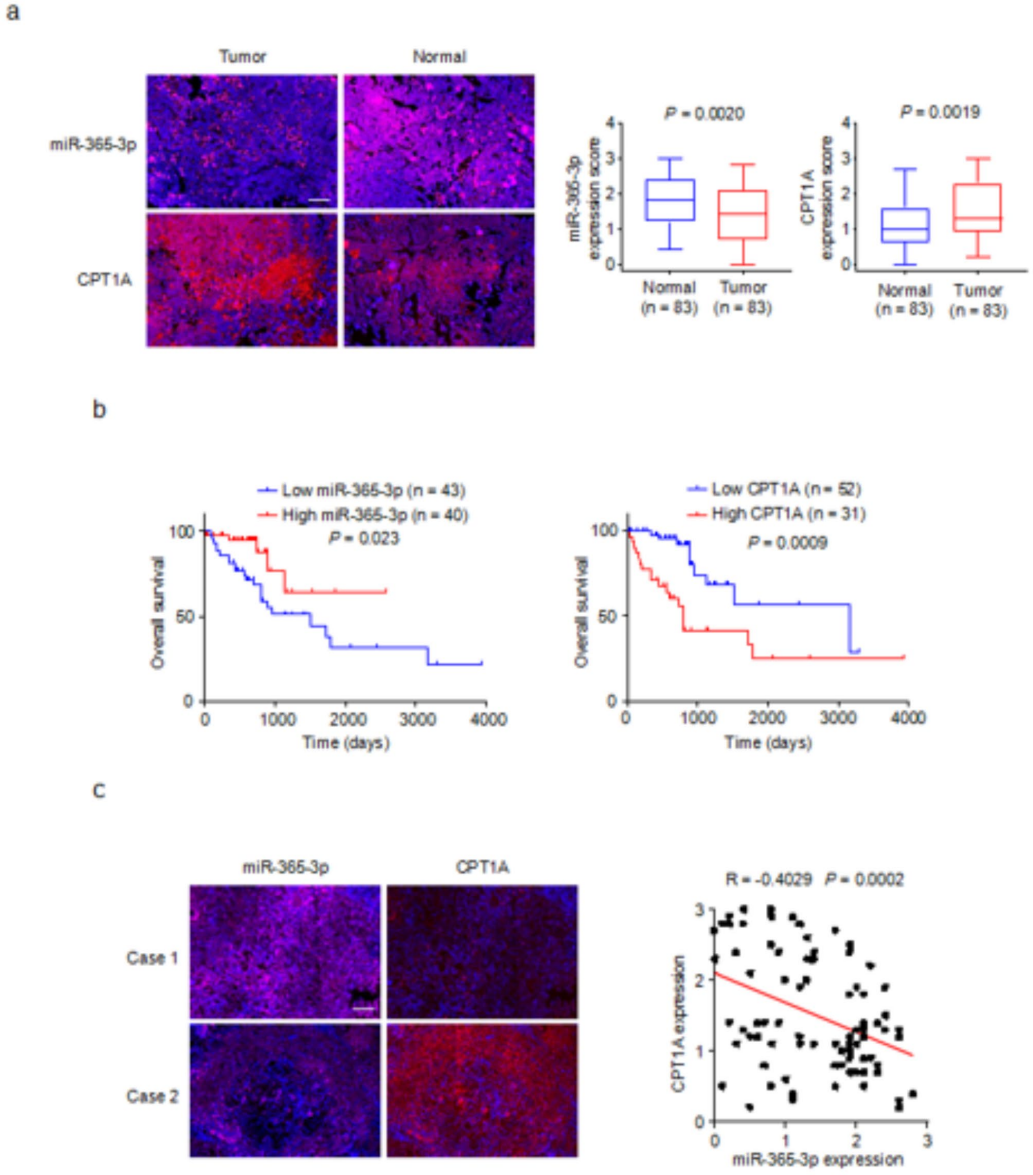
miR-365-3p is negatively correlated with CPT1A expression and predicts clinical outcome in lung cancer patients. (**a**) Representative images of miR-365-3p and CPT1A expression in 83 paired lung tumors and adjacent normal tissues. Scale bar, 100 μm. The expression of miR-365-3p or CPT1A between tumor and normal tissues was compared by Mann-Whitney U test. (**b**)The correlation between miR-365-3p or CPT1A expression and overall survival was determined by Kaplan-Meier survival curves. (**c**)Representative images of miR-365-3p and CPT1A expression in 83 lung cancer tissues. Correlation between miR-365-3p and CPT1A expression was performed by Pearson chi-squared test. Scale bar, 100 μm.

To gain deeper insight into the clinical relevance of miR-365-3p and CPT1A in lung cancer, a comprehensive study involving the collection of 83 pairs of lung cancer tissues and their adjacent normal tissues was undertaken. After collection, the tissues were stained with an miR-365-3p probe and CPT1A antibody by immunohistochemistry. The expression levels of miR-365-3p and CPT1A were then quantitatively evaluated using a scoring method. The results showed that, the miR-365-3p expression was significantly down-regulated in lung cancer tissues compared with that in adjacent normal tissues. Conversely, the expression of CPT1A was markedly up regulated in lung tumor tissues compared with that in normal tissues (Fig. 4a). Pearson’s chi-square test indicated that while miR-365-3p/CPT1A was not associated with most clinical characteristics, miR-365-3p was closely correlated with tumor stage, lymph node status, and pathological stage. Similarly, CPT1A expression was closely associated with tumor stage (Table S3). Additionally, survival analysis highlighted a significant correlation between the expression levels of miR-365-3p and CPT1A and the overall survival (OS) in patients. OS was defined as the interval between the date of diagnosis and the date of death from any cause. Patients with higher miR-365-3p expression tended to have a prolonged OS period, whereas those with elevated CPT1A expression levels experienced a decrease in their OS rate (Fig. 4b). These findings indicate the substantial influence of miR-365-3p and CPT1A expression levels on patient survival outcomes. Building on our initial assessment of miR-365-3p and CPT1A expression levels in lung cancer and adjacent normal tissues, we further investigated the relation within lung cancer. Correlation analysis revealed that miR-365-3p was negatively correlated with CPT1A in the samples of patients with lung cancer, highlighting a clear and consistent pattern across all collected samples (Fig. 4c). In summary, the miR-365-3p/CPT1A axis has substantial clinical significance and offer insights into the potential molecular mechanisms driving lung cancer progression and patient survival outcomes. This axis represents a promising avenue for future research.

## Discussion

Abnormal fatty acid metabolism in tumor cells is closely associated with tumor occurrence, development, invasion and metastasis^24,25^. Fatty acid metabolism includes fatty acid *de novo* synthesis, uptake and oxidation. The greatly enhanced fatty acid *de novo* fatty acid synthesis in tumor cells is recognized as a vital metabolic alteration and is an important indicator of malignancy. FAO produces a large amount of energy necessary for tumor proliferation and is strongly associated with tumor progression in many cancers. Sawyer et al. reported that FAO is increased in ovarian cancer, and that targeting FAO promotes anoikis and inhibits ovarian cancer progression^11^. Wang et al. showed that several genes in the FAO pathway are upregulated in colorectal cancer cells, and that FAO activation increases metastatic capacity^12^. Zeng et al. demonstrated that FAO promotes breast cancer stemness, thereby enhancing tumor metastasis^13^. In our study, FAO played an active role in lung cancer by facilitating tumor development and progression.

CPT1A is the rate-limiting enzyme for FAO, and CPT1A-mediated FAO has been implicated in the progression of many cancers. For example, Pucci et al. reported that CPT1A is involved in breast cancer survival, evasion of cell death, and invasion and has been proposed as a tumor-specific target for anticancer therapeutics^17^. In gastric cancer, CPT1A overexpression activates FAO and promotes cell proliferation, invasion, and EMT process^15^. In prostate cancer, CPT1A knockdown decreased FAO but increased sensitivity to etomoxir through the suppression of AKT kinase and promotion of caspase-3, thereby repressing tumor growth^16^. In the present study, we revealed for the first time the function of CPT1A in lung cancer. CPT1A activates FAO and promotes the cell proliferation and migration of A549 and H1299 cells. Moreover, CPT1A is upregulated in patients with lung cancer, and high expression of CPT1A is associated with shorter OS, indicating a poor prognosis.

It has been reported that miR-365-3p plays a substantial role in various cancers. It targets kruppel-like factor 3 (KLF3) and significantly suppresses the migration, invasion, and chemoresistance of colorectal cancer cells, suggesting that miR-365-3p may be a potential tumor suppressor in colorectal cancer^26^. In oral squamous cell carcinoma, miR-365-3p decreases the migration, invasion, metastasis, and chemoresistance of oral squamous cell carcinoma cells by repressing of keratin 16 (KRT16)^27^. Gao et al. reported that miR-365-3p regulates forkhead box K1 (FOXK1) to inhibit cell proliferation, migration, invasion, and EMT process in breast cancer^28^. MiR-365-3p also acted as a tumor suppressor in gastric cancer and glioma^29,30^. Importantly, the function of miR-365 in lung cancer was controversial. Cao et al. reported that miR-365 inhibited growth, invasion and metastasis of lung cancer by targeting NRP1 expression^31^, and Tong et al. showed that miR-365 inhibited the progression of lung adenocarcinoma through targeting ETS1 and inactivating AKT/mTOR pathway^32^. However, Wang et al. reported that miR-365 promotes lung carcinogenesis by downregulating the USP33/SLIT2/ROBO1 signaling pathway^33^. In our study, we demonstrated that miR-365-3p targetes CPT1A and inhibits its expression. Furthermore, miR-365-3p regulates CPT1A-mediated FAO and decreases the proliferation and migration of lung cancer cells. Among the miRNAs targeting CPT1A, only one study has shown that miR-328-3p targets CPT1A to modulate FAO and repress breast cancer stemness and metastasis^13^. To the best of our knowledge, our study is the first to identify CPT1A as a target gene of miR-365-3p in lung cancer, enriching the understanding of upstream molecules regulating CPT1A. We demonstrated that miR-365-3p not only inhibits FAO in lung cancer, but also decreases lung tumor growth by suppressing CPT1A-mediated FAO. In addition, miR-365-3p is downregulated in patients with lung cancer, and high expression of miR-365-3p is associated with longer OS, indicating a good prognosis. In summary, miR-365-3p was regarded as a tumor suppressor in lung cancer in our study, consistent with the findings of two previously reported studies^31,32^. However, another study reached an opposite conclusion^33^. We speculate that potential reasons for this discrepancy may include variations in cell lines, experimental materials, or technical methods across different laboratories, among other factors.

In conclusion, this is the first study to demonstrate the biological function of the miR-365-3p/CPT1A axis in lung cancer. We found that miR-365-3p targets CPT1A to modulate its expression and inhibit cell proliferation and migration both in vivo and in vitro. In patients with lung cancer, miR-365-3p expression is negatively correlated with CPT1A expression, and both miR-365-3p and CPT1A expression levels predict good and poor clinical outcomes, respectively. There is an urgent need to identify and validate new biomarkers that can improve the accuracy of risk stratification in lung cancer patients. This advancement would help optimize treatment strategies and enhance patient outcomes^34^. Thus, the miR-365-3p/CPT1A axis may be a promising therapeutic target in lung cancer.

## Electronic supplementary material

Below is the link to the electronic supplementary material.

Supplementary Material 1

## Data Availability

The datasets in the current study are available from the corresponding author on reasonable request.
