# Supplementary material for: MiR-365-3p inhibits lung cancer proliferation and migration via CPT1A-mediated fatty acid oxidation
Source: Sci Rep. 2025 Feb 27;15:7076. doi: 10.1038/s41598-025-91665-x (PMC11868381; doi:10.1038/s41598-025-91665-x)

Figure S1

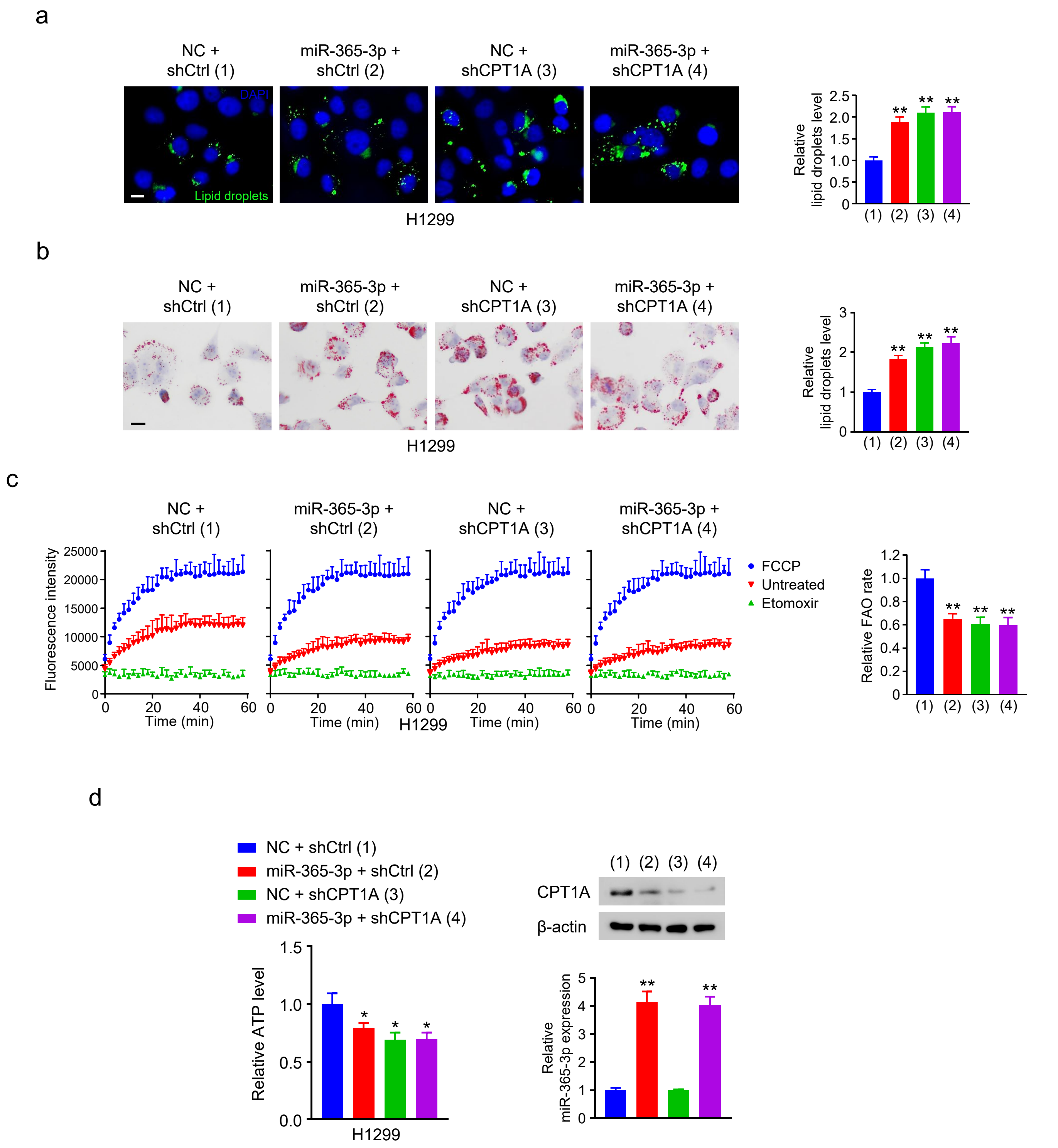

Fig. S1. miR-365-3p suppresses FAO through CPT1A in lung cancer cells.
a Immunofluorescence staining detection of lipid droplets (green) level in H1299 cells stable infected with lentivirus carrying CPT1A shRNA (shCPT1A) or control shRNA (shCtrl) and transfected with miR-365-3p mimics or NC. Scale bar, 10 μm. Histograms show relative lipid droplets level. **b** Oil Red O staining assay of H1299 cells transfected as in (**a**). Scale bar, 10 μm. Histograms show relative lipid droplets level. **c** FAO assay of H1299 cells transfected as in (**a**). Cells treated with FCCP or Etomoxir were used as positive or negative control, respectively. Histograms show relative FAO rate. **d** ATP production assay of H1299 cells transfected as in (**a**). The representative western blot shows the expression of CPT1A. qRT-PCR analysis indicates the expression of miR-365-3p. *, *P* < 0.05; **, *P* < 0.01.

Figure S2

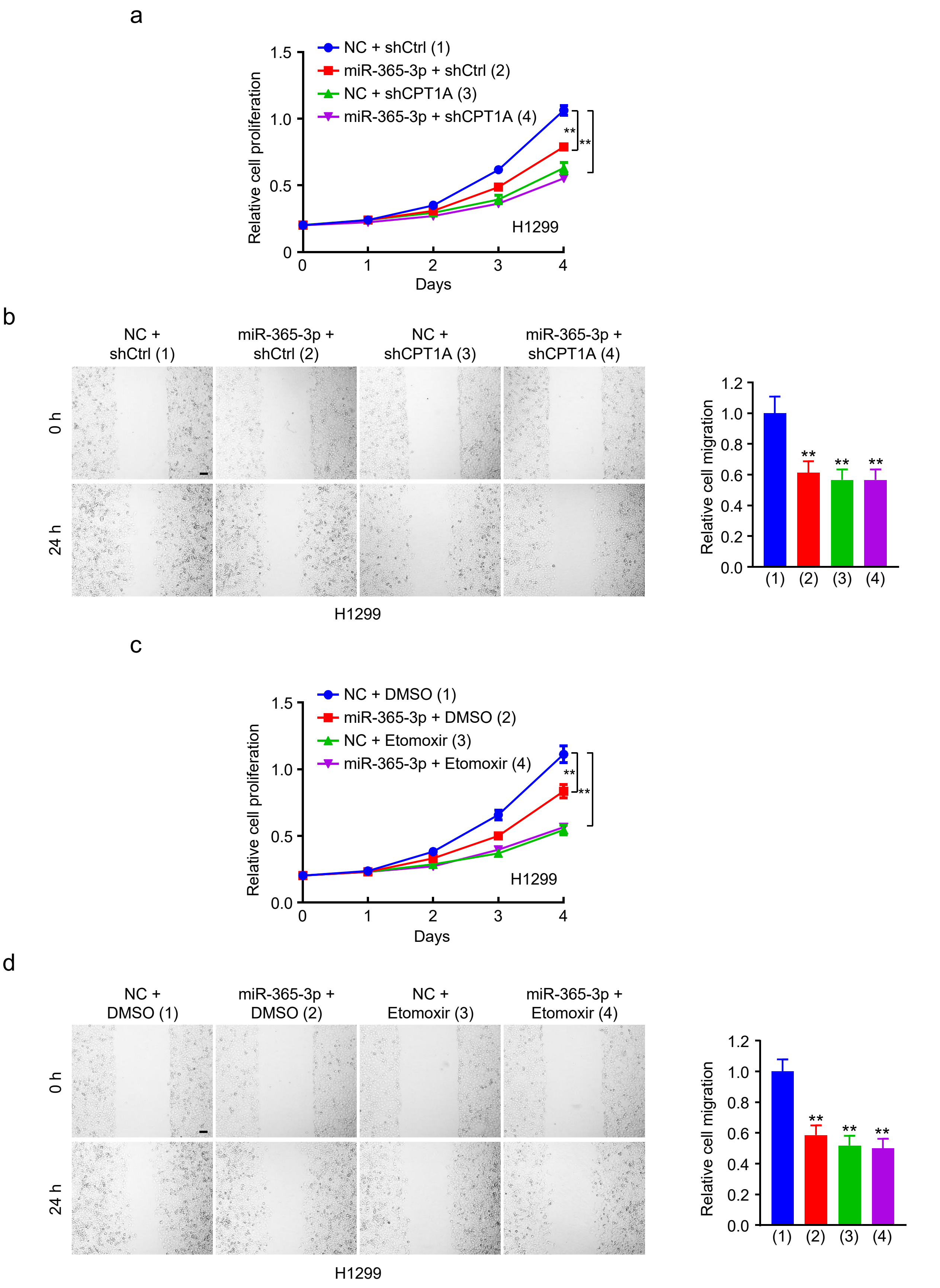

Fig. S2. The miR-365-3p/CPT1A axis regulates lung cancer cell proliferation and migration *in vivo* and *in vitro*.
a Cell proliferation detection of H1299 cells stable infected with lentivirus carrying CPT1A shRNA (shCPT1A) or control shRNA (shCtrl) and transfected with miR-365-3p mimics or NC. **b** Wound healing assay of H1299 cells transfected as in (**a**). Scale bar, 100 μm. Histograms show relative cell migration. **c** Cell proliferation detection of H1299 cells transfected with miR-365-3p mimics or NC and treated with DMSO or Etomoxir (5 μM) as indicated. **d** Wound healing assay of H1299 cells transfected as in (**c**). Scale bar, 100 μm. Histograms show relative cell migration. *, P < 0.05; **, P < 0.01.

Table S1 Clinical characteristics of the patients

No.	Sex	Age	Tumor stage	Lymph node stage	Metastasis stage	Pathological stage
1	Female	84	T1	N0	M0	Stage I
2	Female	62	T1b	N1	M0	Stage IIA
3	Male	61	T1a	N0	M0	Stage IA
4	Male	59	T2b	N0	MX	Stage IIA
5	Male	77	T1	N2	M0	Stage IIIA
6	Male	43	T2	N0	M0	Stage IB
7	Female	79	T3	N1	M0	Stage IIIA
8	Male	59	T2b	N1	MX	Stage IIB
9	Female	71	T2	N2	M0	Stage IIIA
10	Female	59	T4	N2	M0	Stage IIIB
11	Female	60	T2	N0	M0	Stage IB
12	Male	69	T1	N0	M0	Stage IA
13	Male	61	T2	N2	M0	Stage IIIA
14	Female	49	T1	N2	M0	Stage IIIA
15	Female	58	T2b	N0	M0	Stage IIA
16	Male	77	T2a	N1	MX	Stage IIA
17	Male	85	T2b	N0	M0	Stage IIA
18	Female	63	T2a	N0	M0	Stage IB
19	Female	60	T1b	N0	MX	Stage IA
20	Male	52	T3	N2	M0	Stage IIIA
21	Male	55	T2	N2	M0	Stage IIIA
22	Female	66	T2	N1	M0	Stage IIB
23	Female	42	T2a	N0	M0	Stage IB
24	Female	87	T2a	N0	MX	Stage IB
25	Female	70	T1a	N0	MX	Stage IA
26	Female	55	T2a	N0	M0	Stage IB
27	Female	69	T2a	N0	M0	Stage IB
28	Female	45	T1a	N0	M0	Stage IA
29	Male	65	T2	N0	M0	Stage IB
30	Female	62	T1	N0	M0	Stage IA
31	Male	74	T2	N0	MX	Stage IB
32	Male	67	T1b	N0	MX	Stage IA
33	Female	66	T2b	N2	M0	Stage IIIA
34	Male	81	T2a	N0	M0	Stage IB
35	Female	60	T2a	N1	MX	Stage IIA
36	Female	55	T1a	N0	MX	Stage I
37	Male	74	T1b	N0	M0	Stage IA
38	Female	78	T2a	N2	MX	Stage IIIA
39	Female	70	T1a	N0	M0	Stage IA
40	Male	54	T2a	N0	M0	Stage IB

41	Male	57	T1b	N1	M0	Stage IIA
42	Female	73	T1b	N0	M0	Stage IA
43	Male	70	T1b	N0	MX	Stage IA
44	Female	54	T2b	N0	M0	Stage IIA
45	Male	65	T2	N0	M0	Stage IA
46	Female	64	T2a	N0	MX	Stage IB
47	Male	64	T3	N0	M0	Stage IIB
48	Male	82	T2	N0	M0	Stage IB
49	Male	65	T2	N1	M0	Stage IIB
50	Female	59	T2	N0	M0	Stage IB
51	Female	72	T1	N0	M0	Stage IA
52	Female	60	T2a	N0	M0	Stage IB
53	Female	60	T2a	N0	M0	Stage IB
54	Male	60	T1a	N0	MX	Stage IA
55	Male	74	T1a	N0	M0	Stage IA
56	Female	67	T1b	NX	M0	Stage IA
57	Male	74	T2	N1	M0	Stage IIB
58	Male	56	T1b	N0	M0	Stage IA
59	Female	73	T1b	N0	M0	Stage IA
60	Female	51	T2	N1	M0	Stage IIB
61	Male	56	T2b	N0	MX	Stage IIA
62	Male	64	T1b	N0	M0	Stage IA
63	Male	64	T1b	N0	M0	Stage IA
64	Female	75	T1	N0	M0	Stage IA
65	Female	63	T1a	N0	M0	Stage IA
66	Female	56	T1	N0	MX	Stage IA
67	Male	61	T2b	N1	M0	Stage IIB
68	Male	57	T1b	N0	M0	Stage IA
69	Male	52	T2	N0	M0	Stage IB
70	Female	66	T1	N0	M0	Stage IA
71	Female	59	T2	N0	MX	Stage IB
72	Female	47	T4	N3	M0	Stage IIIB
73	Female	52	T2	N1	M0	Stage IIB
74	Male	67	T2	N0	M0	Stage IB
75	Female	54	T2	N0	M0	Stage IB
76	Female	76	T1	N2	MX	Stage IIIA
77	Male	59	T1	N0	MX	Stage IA
78	Female	71	T2	N1	M0	Stage IIB
79	Male	63	T2	N0	MX	Stage IB
80	Male	58	T2	N0	M0	Stage IB
81	Male	75	T1	N0	M0	Stage IA
82	Male	60	T2	N0	M0	Stage IB
83	Male	71	T4	N0	M0	Stage IIIB

Table S2 Prime sequences for shRNA, miRNA mimics, and qRT-PCR

Primer	Sequences (5'→3')
miR-33a-5p mimics	GUGCAUUGUAGUUGCAUUGCA
miR-188-5p mimics	CAUCCCUUGCAUGGUGGAGGG
miR-328-3p mimics	CUGGCCCUCUCUGCCCUUCCGU
miR-365-3p mimics	UAAUGCCCCUAAAAAUCCUUAU
miR-506-3p mimics	UAAGGCACCCUUCUGAGUAGA
miR-365-3p for qRT-PCR	GTCGTATCCAGTGCAGGGTCCGAGGT<br>ATTCGCACTGGATACGACATAAGG
miR-365-3p-F	CGCGTAATGCCCCTAAAAAT
miR-365-3p-R	AGTGCAGGGTCCGAGGTATT
U6-F	CTCGCTTCGGCAGCACA
U6-R	AACGCTTCACGAATTTGCGT
CPT1A-F	TTCCTGGGCGGACGCGCCC
CPT1A-R	CTGCAGAGTTCAAGTGGGCCTG
β -actin-F	TCGTGCGTGACATTAAGGAG
β -actin-R	ATGCCAGGGTACATGGTGGT
CPT1A shRNA	GCCATGAAGCTCTTAGACAAA

Table S3 Correlation of miR-365-3p and CPT1A expression with clinical characteristics

Clinical characteristics	Total cases	miR-365-3p high	miR-365-3p low	P value	CPT1A high	CPT1A low	P value
**Sex**				0.455			0.385
Male	40	21	19		13	27	
Female	43	19	24		18	25	
**Age**				0.492			0.071
≥ 50 years	57	26	31		25	32	
< 50 years	26	14	12		6	20	
**Tumor stage**				0.092			0.112
T1	33	18	15		10	23	
T2	44	21	23		17	27	
T3	3	1	2		2	1	
T4	3	0	3		2	1	
**Lymph node stage**				0.118			0.715
N0	57	30	29		21	38	
N1	13	8	5		5	8	
N2	10	2	8		5	5	
N3	1	0	1		0	1	
**Pathological stage**				0.148			0.413
I	52	26	26		19	33	
II	17	12	5		5	12	
III	13	2	11		7	6	

Original western blots

Figure 1

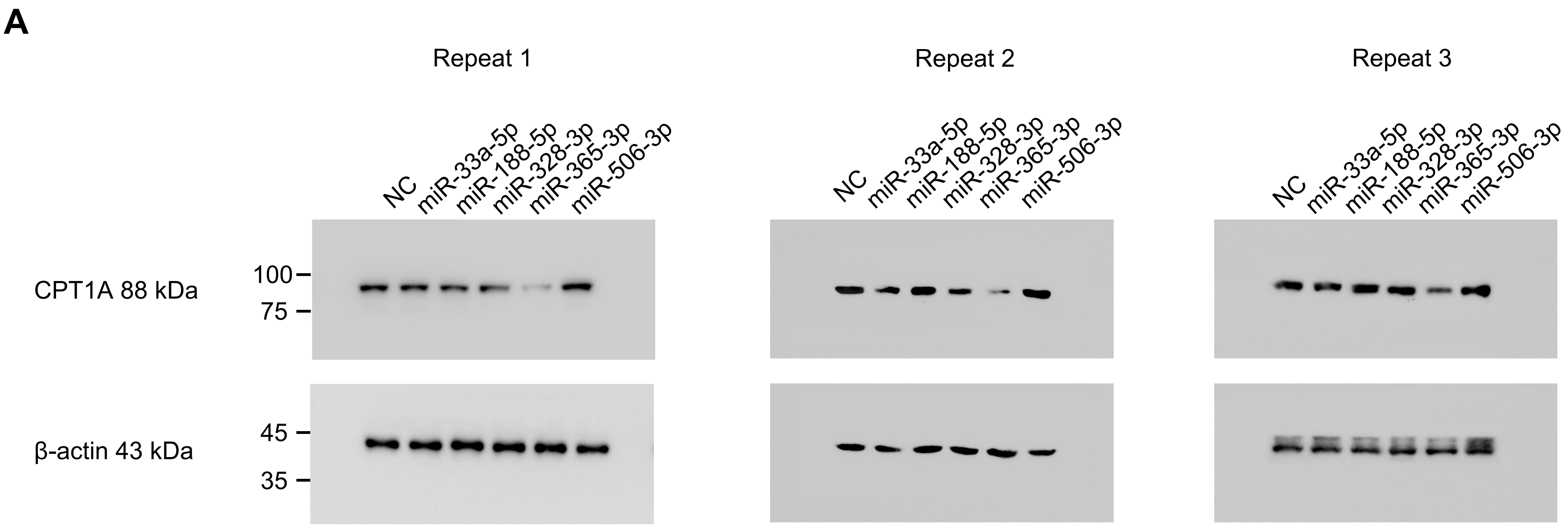

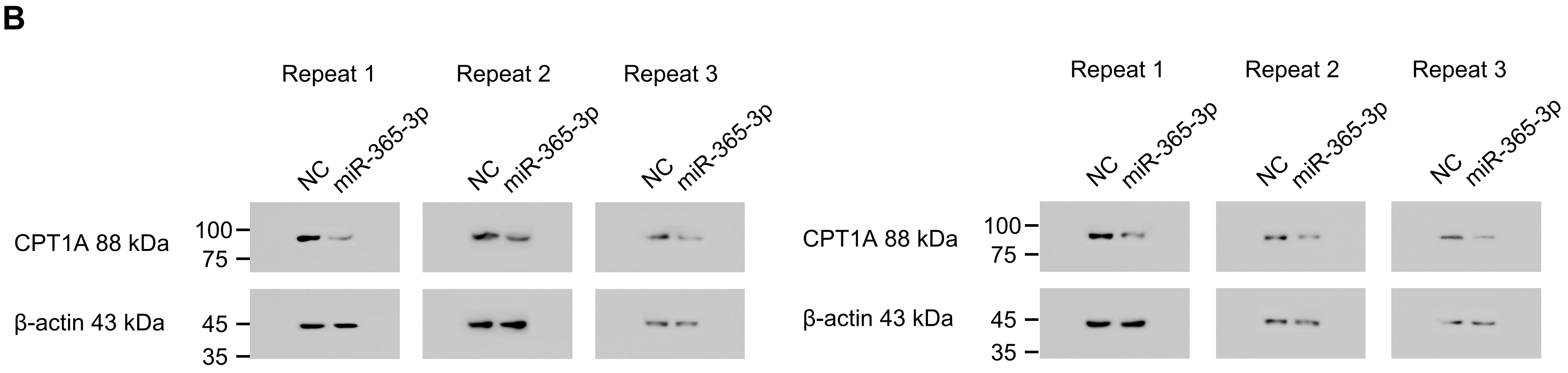

Original western blots

Figure 2

D

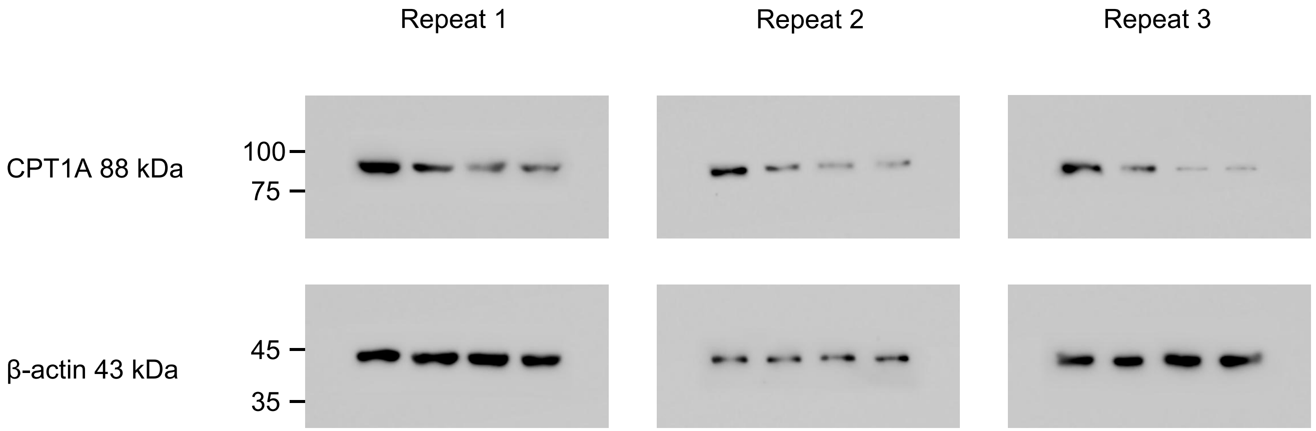

Original western blots

Figure 3

F

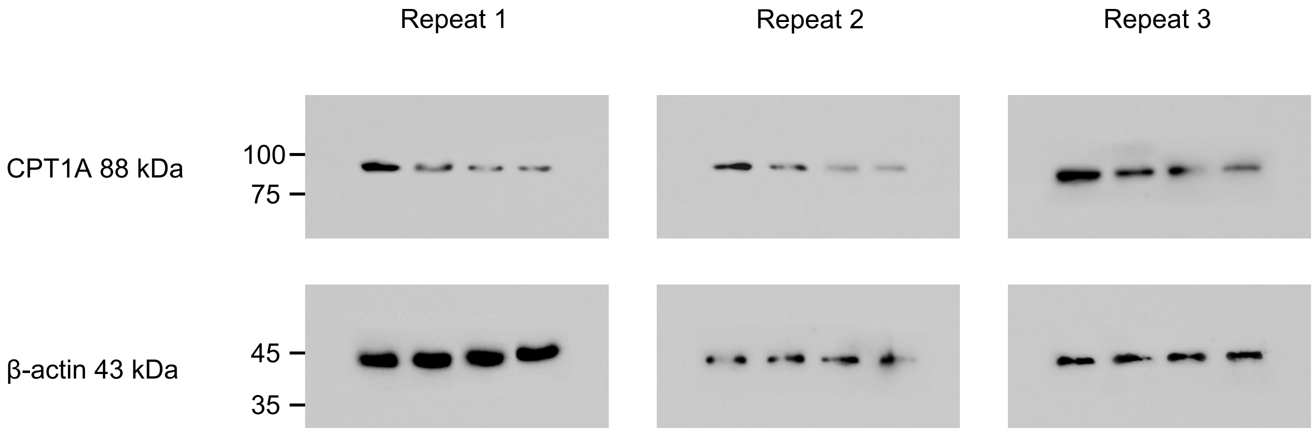

Original western blots

Figure S1

D

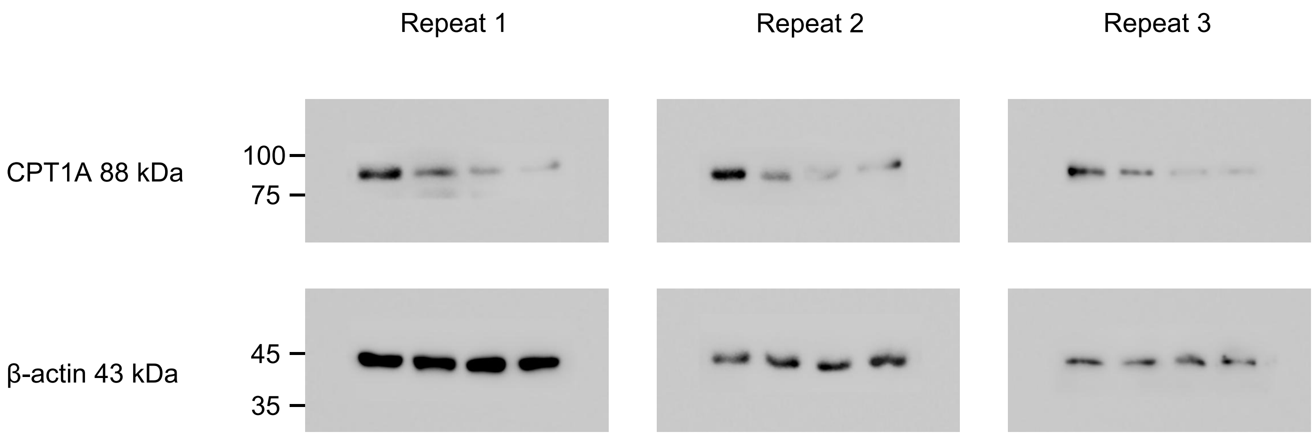

Supplement: Supplementary file 1 — Supplementary Material 1 [file 41598_2025_91665_MOESM1_ESM.pdf]
